# The Task of the New Water Authority

**Published:** 1902-12-27

**Authors:** 


### The Task of the New Water Authority.

It is often only after a bargain has been concluded that a man begins seriously to recognise all the responsibilities which attach to his new purchase, and it is fair to believe that the newly constituted water authority for London will soon find out many little flaws in their new possession which were passed over very lightly while the bargain was in progress. If the London water companies during their long existence had been kept well up to the mark by those placed in authority over them, if the inhabitants of London had ever been so impressed with the importance of a pure water-supply as to have insisted on the companies rectifying at once every imperfection which was discoverable in their appliances, the effect of the Act would have been to transfer the water business to the newly constituted authority as a going concern, fully equipped and in full working order. But this is far from being the case. Under the comparatively lax supervision which has been exercised over the water companies, many things have been done and many things have been left undone, the doing and the omission of which will not be permissible to a public body. We can hardly doubt, for example, that one of the first things which the new authority will have to do will be to relay many miles of pipes, some of which are so near the surface as to be exposed to the action of frost, while others are so leaky as to involve a danger of absorbing infection from the polluted soil in which they lie every time the water is cut off. Nor can we doubt that if rigid rules were enforced as to the rate at which the water is allowed to pass through the filters, a considerable enlargement of the filtering area would at once become necessary, especially so long as from want of storage-capacity (the deficiency of which is notorious) storm water has to be made use of before it has had sufficient time to clear itself by sedimentation. This brings us to the question of storage, and as to this we may refer to the recently issued report of the Local Government Board, which shows how very near the wind the companies have been sailing in regard to the amount of water that they have been abstracting from the Thames. When the rivers are swollen by floods, the quantity taken would never be missed. Even on the average for whole months it sometimes does not amount to much more than 5 per cent. Far otherwise is it, however, in the dry months—in July, August, and September—when, while the rivers are at their lowest, the demand for water among the consumers is greater than ever. For example, in August of last year upwards of 43 per cent. of all the water flowing was abstracted by the companies. It is by their capacity for meeting the demand at such times as these, that we must judge of the rivers' fitness as a source of water-supply.

Even this, however, does not fully show how nearly we have arrived at the limit of the supply to be obtained, except by aid of far greater storage than has so far been provided. To get at this we have to observe what happens on single days. Even in months in which on the average a comparatively small proportion of the water may be abstracted, days occur which show a very different state of affairs. Take, for example, July of last year. In that month only 39 per cent. of the average daily volume of discharge was abstracted, but there were days when that amount was far exceeded, as happened on July 23rd, when of the total flow of 239,000,000 gallons only 84,000,000 gallons passed over the weir at Teddington, all the rest, amounting to more than 155,000,000 gallons, being abstracted for water-supply purposes.

The River Lea is in even worse case than the Thames, and it is clear that these rivers can only be trusted on the condition of a continuance of the system already initiated of building immense storage-reservoirs at an immense expense.

Dr. Thorpe says that the Chelsea, West Middlesex, and Southwark companies, having from 13 to 18 days' storage, delivered the best water; while the Grand Junction Company, with less than three days' storage, delivered water containing most organic impurity. Evidently filtration alone, at any rate as at present conducted, cannot be trusted to secure uniformity of product, and Mr. Perrin says that "excellent as the quality of the filtered water usually is, there are undoubtedly occasions when a better result would have been obtained by longer settlement, and it is hoped that the companies will keep in view the recommendations of the Royal Commission." It has been all very well for water companies working under Acts obtained long ago to ignore the recommendations of a Royal Commission, and for different companies drawing from the same source and serving neighbouring districts to distribute water so different in character as that provided, say, by the Chelsea and the Grand Junction, but such vagaries will not be permitted to a public water-authority. Every district in London will demand a water-supply equal to the best, and to give such a supply vast sums of money will be required to supplement the present plant.

